# Polycystic Ovarian Syndrome: Correlation between clinical hyperandrogenism, anthropometric, metabolic and endocrine parameters

**DOI:** 10.12669/pjms.35.5.742

**Published:** 2019

**Authors:** Ayesha Khan, Nasim Karim, Jahan Ara Ainuddin, Muhammad Faisal Fahim

**Affiliations:** 1 Dr. Ayesha Khan, MBBS. Senior Lecturer & MPhil Student, Pharmacology Department, Bahria University Medical & Dental College, Sailors Street, Adjacent PNS Shifa, Defence Phase II, Karachi, Pakistan; 2 Prof. Dr. Nasim Karim, MBBS, MPhil, PhD, Post-Doc. Head of Department Pharmacology, Pharmacology Department, Bahria University Medical & Dental College, Sailors Street, Adjacent PNS Shifa, Defence Phase II, Karachi, Pakistan; 3 Prof. Dr. Jahan Ara Ainuddin, MBBS, MCPS, FCPS, PhD. Head of Department Obstetrics & Gynecology, DIMC-DUHS, & Consultant Obstetrician & Gynecologist Mamji Hospital, Karachi, Pakistan; 4 Muhammad Faisal Fahim, Msc. Statistics. Statistician, Bahria University Medical & Dental College, Karachi, Pakistan

**Keywords:** PCOS, Hirsutism, F-G score, Correlation, Anthropometric parameters, Metabolic parameters, Endocrine parameters

## Abstract

**Objective:**

To evaluate the correlation between clinical hyperandrogenism-hirsutism assessed by modified Ferriman-Gallwey (F-G) score, anthropometric, metabolic and endocrine parameters among PCOS infertile women.

**Methods:**

This observational study after approval of FRC & ERC of BUMDC was conducted from September 2018-March 2019. It included seventy women aged 20-40 years who presented in infertility clinic of a local Hospital in Karachi. After written informed consent participants were enrolled as per the inclusion criteria of the study and evaluated for cyclical pattern (oligomenorrhoea, amenorrhoea, polymenorrhea), physical (weight, height, BMI), anthropometric, (waist circumference, hip circumference, waist to hip ratio, hirsutism), metabolic (carbohydrate, lipid & protein) and endocrine parameters (serum FSH, LH, LH/FSH ratio, serum testosterone, prolactin and progesterone level). Hirsutism was assessed by visual method through modified F-G score and Pearson correlation was determined between hirsutism and other study parameters.

**Results:**

A positive Pearson correlation is found between hirsutism and body weight, BMI, waist and hip circumference, waist to hip ratio (WHR), very low density lipoprotein, cholesterol, triglycerides and testosterone levels.

**Conclusion:**

Hirsutism has correlation with anthropometric, metabolic and hyperandrgenic disorders in PCOS infertile women as assessed by modified F-G score.

## INTRODUCTION

Polycystic ovary syndrome (PCOS) is an endocrine disease of females commonly present during reproductive life. The pathophysiology of this disease mainly includes chronic anovulation, hyperandrogenemia, and insulin resistance which may present clinically as abnormal uterine bleeding, hirsutism, infertility etc. Prevalence of PCOS varies from 5% to 10 % in reproductive age group females.^1^ The most applicable tool to diagnose PCOS worldwide is revised Rotterdam criteria 2003.^2^

PCOS has multiple origins but exact cause is still unknown. The disease develops due to excessive production of androgens by ovaries along with excessive release of insulin and luteinizing hormone (LH). Hyperinsulinemia causes increase gonadotrophin releasing hormone (GnRH),reverses LH/FSH ratio such as to 3:1 or 2:1, increases level of androgen and decreases production of sex hormone-binding globulin (SHBG).^3^ Insulin resistance associated with excessive insulin level can cause metabolic abnormalities which are characteristic feature of this syndrome.^4^ Obesity, decreased sensitivity of insulin, pre-diabetes, type II diabetes, cardio-metabolic and psychological features such as anxiety, depression etc. are the main metabolic features present in PCOS patients 5,6

Hirsutism is a condition characterized by excessive hair growth in androgen-dependent areas of the body. Hairs become coarse and are distributed over the androgen dependent areas in male pattern. The etiology of hirsutism is mainly classified as: idiopathic, androgenic and nonandrogenic. Eighty percent of the females of reproductive age group, who present to clinical settings with hirsutism, most commonly have PCOS.^7^ It has been documented that about 70-80% of hirsute women have PCOS while non-androgenic factors are relatively rare.^8^

A lot of hair follicles are distributed all over the body surface but their number remains constant throughout individual’s life. The size of follicles and texture of hair can vary in response to various factors and availability of androgen. Thus hirsutism reflects the interaction between concentration of androgen within the circulation and the sensitivity of the hair follicle to androgens. Process of keratinization, increased size of hair follicle, diameter of fiber and the time period of terminal hairs in the anagen phase all depend upon androgen. However, stimulation of hair growth depends on degradation and variation in sensitivity of organs to androgens in circulation and also on insulin resistance.^9^

**Fig.1:**
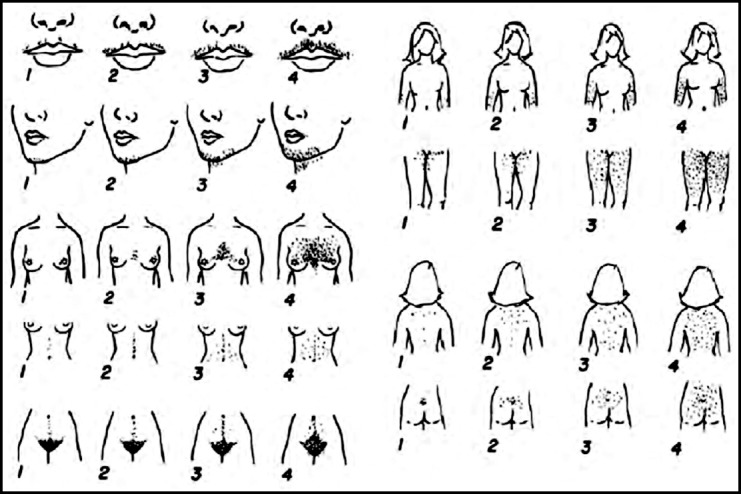
Modified Ferriman-Gallwey Score (mFG score^10^).

The grading of hirsutism was described in 1961 by Ferriman and Gallwey through a simple scoring system. According to this scoring system, terminal hairs of eleven different sites of body are graded from zero-four with a cumulative score of thirty six. However in modified Ferriman and Gallwey (mFG) score number of body sites is reduced to nine after excluding forearm and lower leg. Absence of hirsutism is indicated by a score of less than eight, mild hirsutism is equal to a score of eight-sixteen, moderate is seventeen-twenty four and finally a score more than twenty four indicates severe type of hirsutism.^4^ Fig.1.^10^

Positive correlation was found in most of the studies between hirsutism, anthropometric, metabolic and endocrine parameters present in patients with PCOS. Present study was designed to correlate hirsutism with anthropometric, metabolic and endocrine parameters in local PCOS infertile women as documented local literature is scarce on this aspect of PCOS.

## METHODS

This observational study is a part of an ongoing randomized open prospective clinical trial for MPhil research work. After approval (FRC/BUMDC/Phar/004/) from Faculty Research Committee & Ethical Review Committee (ERC 51/2018) dated: 24-09-2018, of Bahria University Medical & Dental College (BUMDC), the study was conducted from September 2018 to March 2019. It included seventy patients who presented in Infertility clinic of Obstetrics & Gynecology outpatient department of Mamji Hospital Karachi were enrolled. Written informed consent was taken from each participant. Inclusion criteria was: infertile women 20-40 years of age, cyclical problems of oligomenorrhoea /amenorrhoea /polymenorrhea, hirsutism, fasting hyperinsulinemia (>10μU/mL, fasting plasma glucose (<7 mmol/L or 126 mg/dl). Exclusion criteria was: women having primary amenorrhoea, perimenopausal women, concomitant medical problems as diabetes, hepatic or renal insufficiencies, tumors of pituitary, ovary or adrenal gland, women using oral contraceptives / injectables and females undergone oophorectomy/ovarian ablation therapy. All patients were evaluated for:

***(A)Cyclical pattern:*** upon history of patient for infertility (failure to achieve a successful pregnancy after 12 months or more of appropriate, timed unprotected intercourse)^11^ oligomenorrhoea (menstrual cycles greater than 35 or 45 days in length, which in turn translates into 8 or less cycles and 10 or less cycles per year respectively),^12^ amenorrhoea (absence of menstruation for more than three cycles or absence of menstruation for greater than six months),^13^ polymenorrhea (refers to cyclic bleeding that is normal in terms of volume but occurs at too frequent intervals of less than 21 days).^14^

(B) Physical & anthropometric parameters weight, height, BMI, waist circumference, hip circumference, waist/hip ratio, hirsutism).

Body weight was taken on weighing machine without shoes and extra clothing. Height was evaluated by measuring tape. Quetelets Index formula was used to calculate BMI. Quetelets Index formula is:


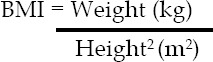


Waist circumference was calculated as the minimum circumference between the iliac crest and the lateral costal margin, whereas the hip circumference was calculated as the maximum circumference over the gluteal region (buttocks), with a measuring tape according to 2003 criteria of WHO.

Waist/hip ratio (waist circumference/hip circumference) was calculated as per Asian criteria waist: hip ratio > or = 0.80 in women was considered indicator of obesity.^15^ Hirsutism was detected by using modified Ferriman - Gallwey score. The method to assess hirsutism by the scoring of terminal hairs in nine body areas, named upper lip, chin, chest, upper abdomen, lower abdomen, upper back, lower back, thighs & upper arms.^4^

***(C) Venous blood*** was taken after overnight fast of 12-14 hours for determination of ***(I) Metabolic parameters:*** (a) Carbohydrate Metabolism (fasting serum glucose, fasting serum insulin level, glucose/insulin ratio)(b) Lipid Metabolism (high density lipoprotein, low density lipoprotein, very low density lipoprotein, cholesterol, triglyceride, (c)Protein Metabolism (serum hs-CRP) and ***(II) Endocrine parameters:*** (serum FSH, LH, LH/FSH ratio, serum testosterone, prolactin and progesterone level).

***(D) Pearson correlation*** between Ferriman-Gallwey score for hirsutism, anthropometric, metabolic and endocrine parameters was applied to evaluate the positive or negative relationship in terms of significance.

### Sample size & Statistical analysis:

Sample size was calculated by “Comparing Two Means” on www.Openepi.com. Frequencies and percentages are calculated for categorical data while mean with standard deviation is documented for continuous variables. Pearson correlation is evaluated between hirsutism and continuous variables and P < 0.05 is considered significant. Data analysis is performed by statistical software package SPSS version 23.

## RESULTS

Physiological, anthropometric, metabolic (carbohydrate, lipid and protein metabolism) and endocrine parameters are sown in Table-I. Mean weight, BMI, WHR, modified Ferriman-Gallwey scoring, fasting serum insulin, low density lipoproteins, triglycerides, hs-CRP and testosterone levels are found to be higher than the standard cut off values. Whereas fasting serum glucose level is towards the higher side. Serum FSH & LH ratio is reversed with LH level almost three times more than the FSH level. Serum progesterone level is well below the normal ovulation range indicating anovulation.

**Table I:** Parameters in study patients (N=70).

A)Physiological & Anthropometric Parameters	Mean ± SD
Age (Years)	28.71±5.237
Weight (kg)	71.49±10.75
Height (m)	1.587±.0410
BMI (Kg/m^2^)	28.45±4.178
Waist Circumference (cm)	92.05±12.831
Hip Circumference (cm)	109.11±10.044
WHR	0.84±.0517
Modified Ferriman-Gallwey Scoring System	9.07±1.753
***B)Metabolic Parameters***	
***1.Glucose Metabolism***	
Fasting serum glucose(mg/dl)	99.31±8.823
Fasting serum insulin(μIU/ml)	13.97±5.492
***2.Lipid Metabolism***	
High density lipoprotein(mg/dl)	42.74±2.019
Low density lipoprotein(mg/dl)	111.74±24.63
Very low density lipoprotein(mg/dl)	36.61±18.70
Serum Cholesterol(mg/dl)	178.46±32.07
Serum Triglyceride(mg/dl)	162.97±52.88
***3.Protein Metabolism***	
Serum hs-CRP (mg/L)	6.01±1.827
***C)Endocrine Parameters***	
Serum FSH(mIU/ml)	3.974±1.248
Serum LH(mIU/ml)	10.81±2.805
LH/FSH	2.885±.235
Serum Testosterone(ng/dl)	78.57±18.27
Serum Prolactin(ng/ml)	17.99±9.276
Serum Progesterone(ng/ml)	1.312±.5635
Serum TSH(μIU/ml)	1.898±.9675

Values represented as mean + standard deviation. BMI: body mass index, WHR: Waist-hip ratio, LH: Luteinizing hormone, FSH: Follicle stimulating hormone.

**Fig.2:**
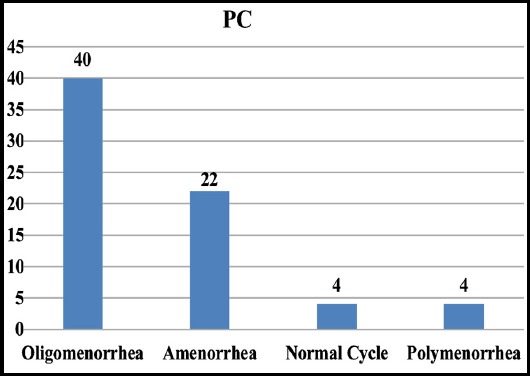
Cyclical pattern.

As regards the cyclical pattern majority 40 (57.14%) patients had oligomenrrhea followed by 22 (31.43%) patients with amenorrhea Fig.2 and Table-II. Mild hirsutism was found in 59 patients, 01 had moderate while 10 had normal distribution of hairs.

**Table II:** Cyclic pattern in study patients (N=70).

Cyclic pattern	Numbers of patients	Percentage
Oligomenorrhea	40	57.14%
Amenorrhea	22	31.43%
Normal cycle	4	5.714%
Polymenorrhea	4	5.714%

Total	70	99.998

Pearson correlation between clinical hyperandrogenism (as per modified Ferriman-Gallwey score) anthropometric, metabolic and endocrine parameters was determined. A positive correlation is found between hirsutism and body weight, BMI, waist and hip circumference, WHR, very low density lipoprotein, cholesterol, triglycerides and testosterone levels Table-III.

**Table III:** Pearson correlation between Ferriman-Gallwey score and anthropometric, metabolic and endocrine parameters (N=70).

Variable	Correlation “r”	P-value
Age	0.020	0.872
weight	0.546	0.000
Height	0.250	0.037
BMI	0.501	0.000
waist circumference	0.468	0.000
Hip circumference	0.475	0.000
WHR	0.397	0.001
Fasting Serum Glucose	0.040	0.741
Fasting Serum Insulin	0.102	0.401
High Density Lipoprotein	0.075	0.538
Low Density Lipoprotein	0.166	0.171
Very Low Density Lipoprotein	0.339	0.004
Serum Cholesterol	0.430	0.000
Serum Triglyceride	0.346	0.003
Serum hs-CRP	0.130	0.284
Serum FSH	-0.108	0.373
Serum LH	-0.199	0.098
LH/FSH	-0.076	0.534
Serum Testosterone	0.797	0.000
Serum Prolactin	-0.042	0.732
Serum Progesterone	0.097	0.422
Serum TSH	0.205	0.089

Significant p <0.05. BMI: body mass index, WHR: Waist-hip ratio, hs-CRP: high sensitivity C-reactive protein, LH: Luteinizing hormone, FSH: Follicle stimulating hormone, r: earson correlation coefficient.

## DISCUSSION

The syndrome of polycystic ovaries is a widespread endocrine disease found in females during reproductive age. The underlying cause of this disease includes chronic anovulation, increased androgen level in the blood, and decreased sensitivity to insulin leading to abnormal uterine bleeding, hirsutism, and infertility. Prevalence of PCOS in reproductive-aged women varies from 4% to 7 %.^2^ Mean age of patients in our study was 28.71±5.237 years which is coinciding with the finding of Usmani and collegues.^16^

International BMI cut-off point as recommended by WHO for obesity is ≥25 kg/m^2^. As per this criteria our patients were obese with a mean BMI of 28.45±4.178.^17^ Hirsutism is found in 70% of women with PCOS.^18^ Approximately 80% to 85% of women with hyperandrogenism clinically have PCOS.^19^ We used hirsutism score as a diagnostic criteria of clinical hyperandrogenism and 85% (60 out of 70) patients in our study had hirsutism.

PCOS women have increased secretion of LH pulse frequency and also have increased level of LH in comparison to FSH in circulation that is the ratio between FSH and LH reverses. There is commonly oligomennorrhea followed by amenorrhea.^20^ Our study results coincides with these findings as 40 (57.14%) out of 70 patients had oligomenrrhea followed by 22 (31.43%) patients with amenorrhea. LH level was almost three times more than the FSH.

Excess of insulin cause anovulation, immature luteinisation of follicles, arrest the development of follicles, which is represented clinically by fasting hyperinsulinemia and low level of serum progesterone accounting for anovulatory infertility.^21^ The same is evident from our study data.

Aswini has documented in her study, waist circumference ≥88 cm, HDL cholesterol <50 mg/dl and serum TG ≥150 mg/dl.^22^ These findings are in favor of our study however we also have found very low density lipoproteins higher than the normal values in our study group.

According to Joint Interim Statement (JIS) criteria, meeting at least 3 of the criteria (WC ≥ 80 cm, FPG ≥100 mg/dl, TG ≥150 mg/dl, HDL-C W< 50 mg/dl) is diagnostic of metabolic syndrome.^23^ Since our patients met all four criteria so they had metabolic syndrome, which is part and parcel of PCOS.

Sales et al.^24^ have documented a positive Pearson correlation between F-G score, BMI and abdominal circumference. This indicates that obesity and adiposity in abdominal area correlates with hirsutism. F-G score may be a utilized as a tool to evaluate the association between hyperandrogenism and cardiovascular risk factors that is the lipid level in PCOS women. A positive correlation is found in our study between hirsutism and body weight, BMI, waist and hip circumference, WHR, very low density lipoprotein, cholesterol, triglycerides and testosterone levels, as per statistically significant evaluation by Pearson correlation. This is coinciding with the finding of Sales et al.^24^ However they have found a positive correlation with insulin level and a negative correlation with testosterone level which is in contrast to our results. This may be due to difference in study populations on geographical basis. Negative correlation was found in our study between F-G score and serum hs-CRP which is coinciding with the finding of Ganie et al.^25^

### Limitations of study

1. This is a single centre study.
2. Free testosterone and DHEAS have not estimated due to budget constraints.

## CONCLUSION

Hirsutism has correlation with anthropometric, metabolic and hyperandrogenic disorders in PCOS infertile women as assessed by modified F-G score. Modified Ferriman-Gallwey score may be used as a marker to evaluate these disorders in PCOS infertile women on account of existence of positive correlation between hirsutism and them.

## RECOMMENDATIONS

Multicentric studies with large sample size in our local population may be undertaken to authenticate our findings and to generate data at the level of Pakistan.
